# A rare presentation of perforated carcinoma of colon as an anterior abdominal wall abscess—a case report and review of literature

**DOI:** 10.1093/jscr/rjab344

**Published:** 2021-11-08

**Authors:** Sunil Basukala, Narayan Thapa, Bikash Bahadur Rayamajhi, Bikram Basukala, Pankaj Mandal, Bibek Karki

**Affiliations:** Department of Surgery, Nepal Army Institute of Health Science (NAIHS), Kathmandu 44600, Nepal; Department of Surgery, Nepal Army Institute of Health Science (NAIHS), Kathmandu 44600, Nepal; Department of Surgery, Nepal Army Institute of Health Science (NAIHS), Kathmandu 44600, Nepal; Department of Surgery, Nepal Army Institute of Health Science (NAIHS), Kathmandu 44600, Nepal; Department of Surgery, Nepal Army Institute of Health Science (NAIHS), Kathmandu 44600, Nepal; Department of Radiodiagnosis, Nepal Army Institute of Health Sciences (NAIHS), Kathmandu 44600, Nepal

## Abstract

Colorectal cancer progresses without any symptoms early on or those clinical symptoms are very discrete and so are undetected for long periods of time. Complicated colorectal carcinoma has several symptoms, the most common being bleeding and obstruction. Occasionally, it will cause perforation, which carries a worse prognosis. It is rare for a carcinoma colon to present as abscess of the anterior abdominal wall that forms as a result of direct invasion and perforation of the colon by cancer. We hereby report an unusual case of perforated colon carcinoma presented as an abscess infiltrating the abdominal wall.

## INTRODUCTION

Colorectal cancer is the third most common cancer and the second leading cause of death in the USA. Approximately 140 000 new cases are diagnosed each year, and almost 55 000 people die from the disease each year [1, 2]. Carcinoma of the colon has the ability to mimic any abdominal disease with a wide spectrum of presentations [3]. Perforation of colorectal cancer is rare as its incidence is 2.6–7.8%. There can be either direct perforation into the peritoneal cavity or local perforation forming an abscess or fistula [4–6]. Direct invasion of transverse colon adenocarcinoma into the abdominal wall is rarely encountered [7]. We hereby report a case of perforated colon carcinoma presented as an abscess infiltrating anterior abdominal wall.

## CASE REPORT

A 63-year-old female patient with diabetes mellitus presented to the emergency department with the complaints of a painful swelling over the right lower abdominal pain for 4 days. It was insidious in onset and was associated with fever, nausea and reduced appetite. She did not have jaundice and hematemesis. However, she gave history of intermittent constipation with occasional bleeding per rectum (PR), abdominal discomfort and sense of incomplete evacuation after defecation for past 6 months. She had undergone an open laparoscopic cholecystectomy 1 year ago for symptomatic gall stone disease and her postoperative period was uneventful. She also gave history of significant weight loss for past 4 months.

**Figure 1:**
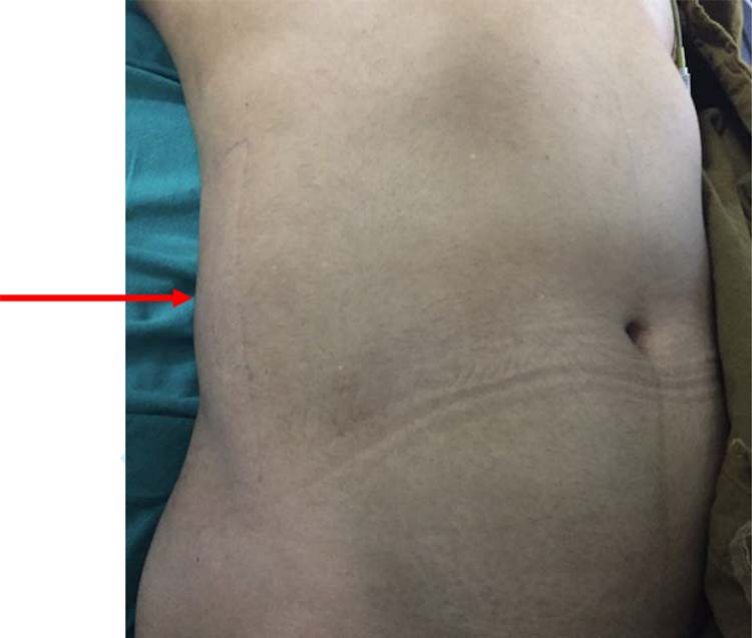
Swelling noted on the postero-lateral aspect of the abdominal wall.

On her physical examination, she was pale and malnourished. She was afebrile, tachycardia and tachypnea were present, and she was in acute distress due to pain. On examination of the abdomen, there was a tender fluctuant area over the right lower abdomen, predominantly in the subcutaneous and muscular plane. It measured around 6 × 8 cm with the erythema of the skin and surrounding induration (Fig. 1). There was no sinus or discharge from the skin. The rest of the abdomen was soft and nontender. On rectal examination, there was normal tone and finger stained with stool with no palpable mass noted. Laboratory investigation revealed an elevated white blood cell count of 16.5 (normal range 4.0–10.8 × 103 per liter) with neutrophils, hemoglobin was below normal limit of 7 mg/dl (normal range 12–16 g/dl) and albumin 2.6 (normal range 3.4–4.8 g/dl). The level of carcinoembryonic antigen was highly elevated.

**Figure 2:**
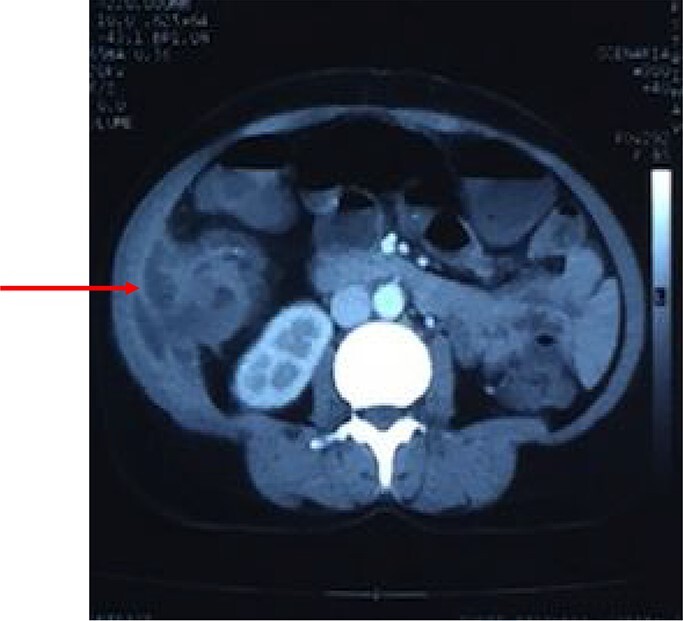
Computed tomography of the abdomen showing perforation of the tumor through muscles of lateral abdominal wall with abscess formation as seen on CT scan.

After resuscitation with intravenous fluids, administration of broad-spectrum intravenous antibiotics and intravenous analgesics, a contrast-enhanced computed tomography (CECT) scan was done to further characterize the lesion. CECT of abdomen showed large abscess extending from the subcutaneous and intermuscular planes over the right iliac fossa and breaching the peritoneum. It showed heterogeneous wall thickening of the proximal part of the ascending colon with loss of mural stratification and causing luminal narrowing, measuring 4.8 × 5.1 × 4.5 cm with surrounding inflammatory changes with adjacent well-defined collection 11.3 × 4.5 × 10.9 cm and few air pockets and infiltrating the right lower abdominal wall (Fig. 2). Pus aspirated from the right abdominal swelling and was sent for culture and sensitivity. Patient was taken up for diagnostic laparoscopy and proceeded.

**Figure 3:**
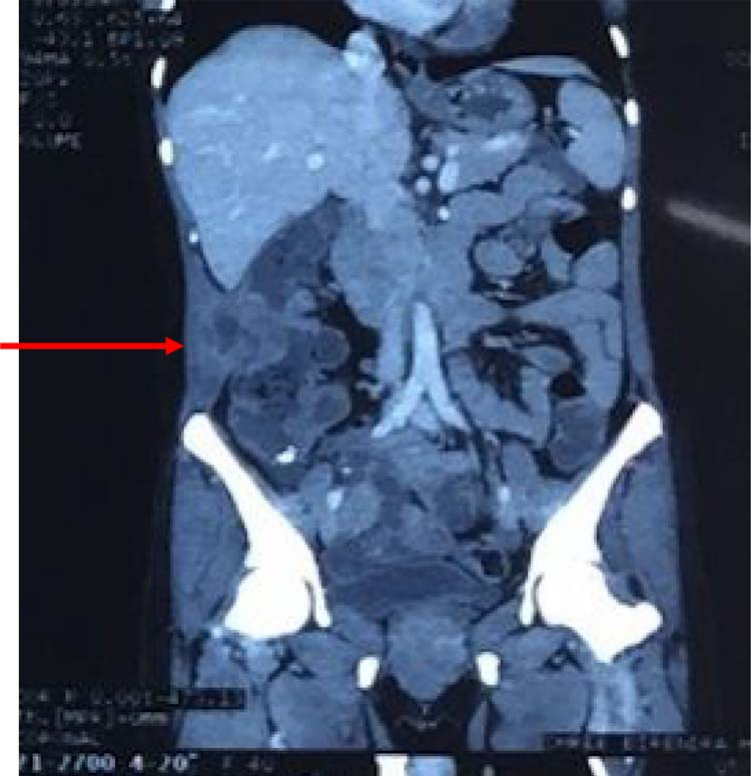
Coronal reconstruction of CT shows mass extending from colon and invading into anterior abdominal wall. Arrows indicate the point of exit of mass through the abdominal wall musculature.

During laparoscopy, a right-sided colonic mass adherent to the lateral abdominal wall with infiltration of the subcutaneous tissue by the tumor was noted (Fig. 3).

**Figure 4:**
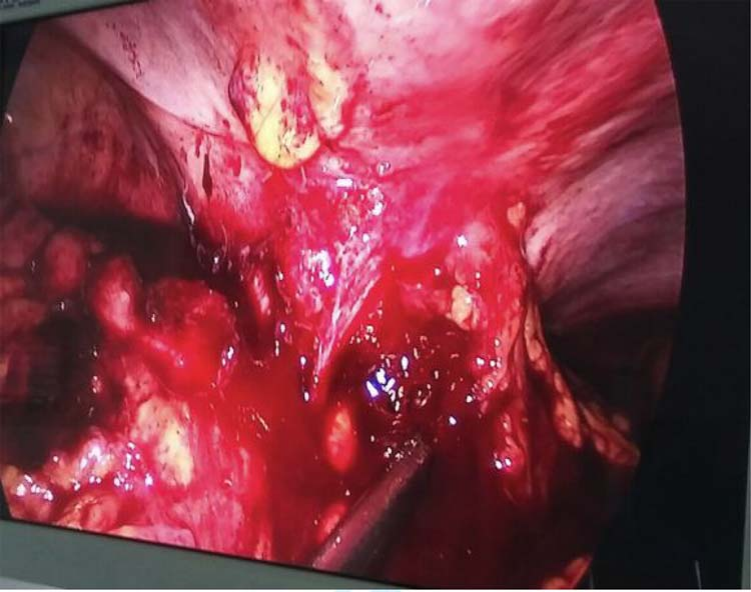
Diagnostic laparoscopy showing ascending colon adherent to anterior abdominal wall.

Ascending colonic cancer complicated by an anterior abdominal wall abscess was diagnosed. Exploratory laparotomy was performed and a right colonic mass ~9 × 8 cm in diameter was found, which was widely adherent to the lateral abdominal wall, with extension through a fistulous track into the subcutaneous tissue (Fig. 4). There was no distant metastasis. Extended right hemicolectomy and lymph node dissection was performed with limited resection (*en bloc*) of the involved part of the lateral abdominal wall using a closure technique.

The patient had an uneventful postoperative recovery. The patient was discharged on the 14th postoperative day with a healthy wound. The patient was referred to Department of Medical Oncology for further adjuvant therapy. Consent was obtained from the patient for her case to be reported.

## DISCUSSSION

About 15% of patients with carcinoma of the large intestine present with surgical emergencies such as perforation and obstruction. The incidence of perforated colorectal cancer ranges from 3 to 10%. Colorectal cancers have the tendency to spread locally, and advanced cancers frequently show direct invasion to adjoining organs and peritoneal dissemination [5–8]. Carcinoma of the colon has the ability to mimic any abdominal disease with a wide spectrum of presentations. Direct invasion of transverse colon adenocarcinoma into the abdominal wall is rarely encountered [6]. Locally advanced colon cancers with direct invasion into adjacent organs or that spreads along the tissue planes may result in formation of abscesses in unusual locations such as the abdominal wall, and sometimes, this can be the first presentation. Abscess formation arising from colon cancer is a rare complication that occurs in 0.3–4% of the cases [9].

When encountering such a case, early diagnosis, appropriate drainage and definitive management may reduce patient morbidity and mortality rates, especially if the patient was in poor condition in which elimination of the source of sepsis may be lifesaving [7]. Confirming a diagnosis of an underlying colon cancer preoperatively poses a surgical challenge. Inaccurate diagnosis without the recognition of the underlying malignancy may lead to incomplete treatment. The conventional investigations like colonoscopy may be inadequate, as it cannot demonstrate abnormalities beyond the lumen of the colon. Repeat biopsies had shown benign lesions, nonspecific pathology reports. Hence, a structured evaluation with computerized tomography (CT) of the abdomen is usually required. CT scans have been reported to be useful for assessing patients with colorectal cancer and inflammatory disease of the abdominal wall [10]. The CT scan in our case showed the presence of malignancy as well as an abscess. Occasionally, a diagnostic laparoscopy or exploratory laparotomy maybe required to ascertain the diagnosis and get a definitive pathological and microbiological sample for diagnosis [4–6], as done in our case.

Once abdominal wall involvement with colon cancer forming an abdominal wall abscess is detected, the existence of cancer cells is highly likely in both the abdominal wall penetrated by the fistula and the wall of the abscess. In such cases, the most appropriate curative procedure would be an *en bloc* excision of the full thickness of the anterior abdominal wall, including the abscess. Complete resection of the malignant colon tumor and the abscess wall is the preferable method. Unless surgery is contraindicated by the patient’s general condition, percutaneous aspiration and drainage by indwelling catheter combined with broad-spectrum antibiotics may be applied without further diagnostic work up [11, 12]. Radical resection is a challenging procedure for surgeons because, in the presence of local invasion, multivisceral resection is required. Although radical resection is essential in patients for cure; unfortunately, most of patients exhibit bad general conditions. Abdominal wall reconstruction is necessary in terms of resection of the abdominal wall muscles and fascia. Primary repair of abdominal wall often fails owing to high tension, and the failure rate of the primary repair may reach up to 50%. On the other hand, large abdominal wall defects related to full-thickness resection of the invasive malignant tumors are not recommended to be closed by primary sutures [13].

## CONCLUSION

A differential diagnosis of carcinoma colon should be considered when an elderly patient presents with abdominal wall abscess accompanied by bowel symptoms such as altered bowel habits or per rectal bleeding, even if there are no other significant clinical symptoms and a thorough investigative work up is required to confirm the diagnosis, to avoid untimely delay in treatment and reduce morbidity and mortality among the patients.
